# Impact of COVID-19 on child malnutrition, obesity in women and household food insecurity in underserved urban settlements in Sri Lanka: a prospective follow-up study

**DOI:** 10.1017/S1368980021001841

**Published:** 2021-04-27

**Authors:** Renuka Jayatissa, Himali P Herath, Amila G Perera, Thulasika T Dayaratne, Nawmali D De Alwis, Hiyare Palliyage Laksiri K Nanayakkara

**Affiliations:** 1 Department of Nutrition, Medical Research Institute, Sir Danister De Silva Mawatha, Colombo 08, Sri Lanka; 2 Independent Consultant

**Keywords:** COVID-19, Wasting, Stunting, Overweight, Obesity, Food security

## Abstract

**Objectives:**

To determine changes and factors associated with child malnutrition, obesity in women and household food insecurity before and after the first wave of COVID-19 pandemic.

**Design:**

A prospective follow-up study.

**Setting:**

In 2019, the baseline Urban Health and Nutrition Study 2019 (UHNS-2019) was conducted in 603 households, which were selected randomly from 30 clusters to represent underserved urban settlements in Colombo. In the present study, 35 % of households from the UHNS-2019 cohort were randomly selected for repeat interviews, 1 year after the baseline study and 6 months after COVID-19 pandemic in Sri Lanka. Height/length and weight of children and women were re-measured, household food insecurity was reassessed, and associated factors were gathered through interviewer-administered questionnaires. Differences in measurements at baseline and follow-up studies were compared.

**Participants:**

A total of 207 households, comprising 127 women and 109 children were included.

**Results:**

The current prevalence of children with wasting and overweight was higher in the follow-up study than at baseline UHNS-2019 (18·3 % *v.* 13·7 %; *P* = 0·26 and 8·3 % *v.* 3·7 %; *P* = 0·12, respectively). There was a decrease in prevalence of child stunting (14·7 % *v.* 11·9 %; *P* = 0·37). A change was not observed in overall obesity in women, which was about 30·7 %. Repeated lockdown was associated with a significant reduction in food security from 57 % in UHNS-2019 to 30 % in the current study (*P* < 0·001).

**Conclusions:**

There was an increase in wasting and overweight among children while women had a persistent high prevalence of obesity. This population needs suitable interventions to improve nutrition status of children and women to minimise susceptibility to COVID-19.

The COVID-19 pandemic threatens the nutrition and food security of millions of people, many of whom are already suffering^(1)^. This pandemic has progressively aggravated issues of global hunger and malnutrition. Pre-existing social, economic and political inequalities have been exposed and exacerbated by the COVID-19 pandemic^(2)^. Furthermore, several COVID-19 response measures such as contact tracing, self-isolation, social distancing and lockdown of communities have led to unhealthy diets and physical inactivity^(1)^. There is a significant concern at a global level as to whether the COVID-19 responses have led to a negative impact on nutritional status of children and women and whether it will worsen with time^(1)^. Additionally, it suggests that economic deterioration, food insecurity and interruption of routine nutrition programmes could increase the prevalence of wasting by 10–50 % with an increase in child mortality^(1)^.

The first COVID-19 case among Sri Lankans was reported on 11 March 2020^(3)^. To prevent the spread of COVID-19 infection, the Sri Lankan government imposed a 52-d continuous lockdown period up to 11 May 2020 in the Western province, with varying lengths of lockdown periods imposed on other provinces^(4,5)^. The new regulations caused major disruptions of lives and income of the people living in under-served settlements (USS) who were mainly daily wage earners. People in USS were confined to the limited living spaces in their houses with no access to physical activity. Implementation of lockdown regulations were monitored by the police. The government implemented cash transfer and food distribution programmes targeting households below the poverty line. The first wave of the COVID-19 pandemic was successfully controlled. People engaged in most of their routine activities while adhering to health and safety precautions from July to September 2020, before the second wave started in October.

Before the onset of the COVID-19 pandemic, the national prevalence of child wasting, obesity in women and household food insecurity in Sri Lanka was 15 %, 30 % and 15 %, respectively^(6,7)^. People in underserved urban settlements were already struggling with food insecurity due to pre-existing crises. They were more nutritionally vulnerable than the rest of the country with a higher prevalence of child malnutrition and obesity in women^(8)^. The vast majority live in very small houses with limited sanitary facilities and depend on daily wages obtained through elementary occupations.

In September 2019, a representative sample of 603 households in underserved urban settlements in the Colombo district was assessed to determine the nutrition and health status of children, women and status of household food security^(8)^. It was observed that the prevalence of child wasting, stunting and overweight was comparable to national rates. However, the prevalence of food insecurity and obesity in women was higher than the national averages^(8)^. Restrictions on movement and activity to control the spread of the pandemic have severely affected the livelihood of the underserved urban settlement population. This has resulted in food insecurity and increased vulnerability to malnutrition. Thus, it is imperative to reassess this cohort of households 6 months after the first local case was reported in the country.

The objective of this study was to determine the changes and associated factors of child wasting, stunting and overweight, obesity in women, and household food insecurity before and after the first wave of COVID-19 pandemic in underserved urban settlements in Colombo.

## Methods

We conducted a prospective follow-up study that included the participants of the Urban Health and Nutrition Study 2019 (UHNS-2019) in selected underserved urban settlements in Colombo district^(8)^.

### Study setting and UHNS-2019

Colombo is the commercial capital of Sri Lanka where nearly 11 % of the total population reside. The population density in Colombo is 3417 persons/km^2^, which is ten times the national average of 345·5 persons/km^2(9)^. Most of the poor urban population in Colombo district live in slum and shanty settlements termed USS. USS comprises areas that have a concentration of residential units built on state or private land and is not owned by the residents. While these residential areas have common features of a very high population density and congested housing (with each block averaging 1·5 perches), it is the unrelieved condition of services and infrastructure available to these residents that give its name^(10–12)^.

UHNS-2019 was based on a cohort of 603 households with 2237 inhabitants randomly selected from 30 clusters of USS areas in Colombo. There were three key target groups within the selected households for UHNS-2019: children aged 0–59 months; adult men aged 18–59 years and adult women aged 18–59 years. Baseline data on household characteristics, sociodemographic and livelihood data, food security, access to health services, dietary intake and nutrition status were collected in UHNS-2019^(8)^.

### Sample size calculation

Sample size was calculated to detect a 0·2 difference in weight-for-height-*Z* (WHZ)/height-for-age-*Z* (HAZ) with a standard deviation of difference of 0·8 in the paired *t* test. At 80 % power, assuming a non-response rate of 5 %, required sample size for the children aged under 5 years was 135. For the food security assessment of households by comparing paired proportions, with estimates of proportion of food-secure households in UHNS 2019 at 60 % and current study at 45 %, 196 households were needed at 80 % power and 10 % non-response rate. Due to the second wave of COVID-19, we had to end data collection activities limiting ourselves to 103 children and 207 households. However, post hoc power calculation revealed that the current study had an acceptable power (72 % power for WHZ and 68 % power for the HAZ) in the paired *t* test and >90 % power for comparing paired proportions (food security status).

### Data collection

**Fig. 1:**
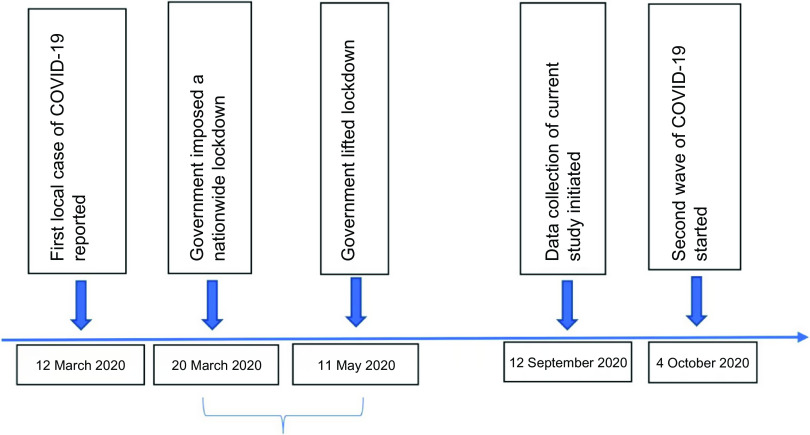
Data collection summary

This follow-up data collection was carried out 1 year after initial recruitment of the cohort and 6 months after the first local COVID-19 case was reported in Sri Lanka as shown in Fig. 1.

This follow-up data collection was carried out 1 year after initial recruitment of the cohort and 6 months after the first local COVID-19 case was reported in Sri Lanka.

Due to the COVID-19 pandemic, follow-up data collection was limited to one-third of the households enrolled in 2019. In this follow-up study, households were randomly sampled from the UHNS-2019 database. Data were collected using a brief, pre-tested, structured questionnaire by direct interview. The data collection tool focused on exposure to COVID-19, change in household income and expenditure following the COVID-19 pandemic and household food insecurity in the preceding 6 months. Self-reported COVID-19 status was assessed during the study. During the interview, respondent was asked whether he/she or anyone in the household was confirmed to have COVID-19 based on the polymerase chain reaction (PCR) test. In these selected households, the same adult woman (aged 18–59 years) and child (aged 0–59 months) who participated in the UHNS-2019 were selected to assess nutritional status.

Food insecurity was assessed using the ‘Food Insecurity Experience Scale (FIES)’ developed by the FAO^(13)^. Participants were instructed to focus on food-related behaviours and experiences of households during the preceding 6 months (COVID-19 pandemic period) when responding to the eight questions in FIES. This questionnaire was composed of a scale that resulted in the categorisation of these households into food-secure, mild (marginal) food-insecure, moderate food-insecure and severe food-insecure groups.

Anthropometric measurements of adult women and children were taken by trained data collectors who followed WHO standard procedures^(14)^. Weight was measured to the nearest 0·1 kg using a calibrated seca weighing scale. Height was measured to the nearest 0·1 cm using a seca stadiometer. Recumbent length (for children < 24 months of age) was measured using an infant measuring board. The same instruments were calibrated daily and used throughout the study.

Indices of nutritional status of children < 60 months were derived using the WHO Anthro Survey Analyzer software and children ≥ 60 months with WHO Anthro Plus software^(15)^. In children aged under 5 years, mild, moderate and severe stunting were defined as HAZ score and WHZ score between −1 sd and −2 sd; −2 sd and −3 sd and below −3 sd, respectively, from the median World Health Organization Child Growth Standards (WHOCGS). Overweight/obesity in children < 60 months was defined as WHZ > +2 sd from the median WHOCGS. In children between 5 and 6 years of age, mild, moderate and severe stunting and wasting were defined as HAZ score and BMI-for-age-sex-*Z* (BAZ) score between −1 sd and −2 sd; −2 sd and −3 sd and below −3 sd, respectively, from the median WHOCGS. Overweight/obesity in children between 5 and 6 years of age was defined as BAZ > +1 sd from the median WHOCGS. Concurrent wasting and stunting (WaSt) were defined as WHZ < −2 SD and HAZ < −2 sd.

In adult women, BMI was calculated (weight in kg/height in m^2^). Underweight, normal, overweight, obesity grade 1, obesity grade 2 and obesity grade 3 (morbid obesity) were defined as BMI < 18·5, 18·5–24·9, 25·0–29·9, 30·0–34·9, 35·0–39·9 and ≥40 kg/m^2^, respectively.

Per capita monthly income was calculated by dividing the monthly household income by total number of household members.

The double burden of malnutrition in households was defined as wasting, stunting overweight of children along with overweight, obesity and underweight of women in the same households^(16)^.

### Statistical analysis

Data were analysed using SPSS (version 22.0, IBM, Inc.) software. The Shapiro–Wilk and Kolmogorov–Smirnov tests were used to test the normality of data. Continuous data were presented as mean and standard deviation when normally distributed. Frequencies and percentages were used to summarise categorical variables. Continuous pre- and post-results were compared using paired *t* test and the categorical, dichotomous pre- and post-variables were compared using the McNemar test. Variables with more than two categories were compared between baseline and follow-up study using McNemar–Bowker test. All tests of significance were two-tailed. A probability level of *P* < 0·05 was used to indicate statistical significance in all analyses.

## Results

A total of 207 households from UHNS-2019 cohort were recruited for the follow-up study. Baseline characteristics of selected households were similar to UHNS-2019 sample except for the proportion of people living in USS since birth, which was significantly higher in the current study compared to UHNS-2019 (Table 1). Mean age and standard deviation of the children in the current follow-up study and at baseline in 2019 were 39 (sd = 16·4) months and 26·4 (sd = 16·3) months, respectively.

**Table 1:** Comparison of baseline characteristics of selected households and the UHNS-2019 cohort

	Follow-up study, September 2020 (*n* 207) %	UHNS-2019, September 2019 (*n* 603) %	*P*
Number of members in the household			
1–3	18·8	20·0	0·71
4–5	52·7	47·9	0·23
≥ 6	28·5	32·1	0·33
Type of the family			
Extended	37·2	39·1	0·63
Nuclear	62·8	60·9	0·63
Residency status			
Living since birth	58·5	48·8	0·02
Permanent but not living since birth	39·6	46·3	0·09
Migrant	1·9	5·0	0·06
Ethnicity			
Sinhalese	45·5	37·8	0·11
Tamil	33·0	36·0	0·53
Muslim	21·5	25·9	0·31
Other	0·0	0·2	0·62

UHNS-2019, Urban Health and Nutrition Study 2019.

### Nutritional status of children and adult women

Nutritional status of 109 children and 127 women in selected households were assessed in this follow-up study. Mean age and standard deviation of the children in the follow-up study was 39 (sd = 16·4) months.

Mean WHZ and HAZ appear to have increased significantly in this study population (Table 2). The proportion of children with moderate to severe wasting was higher, while children with moderate to severe stunting were lower in the follow-up study. However, these differences were not statistically significant (*P* > 0·05). A significant difference was not seen in the distribution of BMI categories in women at baseline and follow-up studies. The prevalence of overweight and obesity among women was more than 60 % in both studies and 2·4 % were morbidly obese. The double burden of malnutrition in households were changed from 13·3 % to 10·8 %, which was not significant.

**Table 2:** Nutritional status of children and women

Nutritional status indicator	Follow-up study, September 2020			UHNS-2019, September 2019			*P*
	*n*	Mean	sd	*n*	Mean	sd	
Children (*n* 109)							
WHZ	109	−0·66	1·35	109	−0·90	1·09	0·02*
HAZ	109	−0·62	1·25	109	−0·78	1·24	0·009*
	*n*	%	95 % CI	*n*	%	95 % CI	
Moderate to severe wasting	20	18·3	11·9, 25·7	15	13·7	7·3, 20·2	0·26†
Moderate to severe stunting	13	11·9	6·4, 18·3	16	14·7	7·4, 21·1	0·37†
Overweight or obese	9	8·3	4·4, 15·0	4	3·7	1·4, 9·1	0·12†
Concurrent wasting and stunting	6	5·5	2·1, 11·6	6	5·5	2·1, 11·6	1†
Women BMI categories (*n* 127)							
Underweight	4	3·1	1·2, 7·9	5	3·9	1·7, 8·9	0·4‡
Normal	33	26·0	19·1, 34·2	34	26·8	19·8, 35·1	
Overweight	51	40·2	32·0, 48·9	51	40·2	32·0, 48·9	
Obesity grade 1	24	18·9	13·0, 26·6	22	17·3	11·7, 24·8	
Obesity grade 2	12	9·4	5·5, 15·8	12	9·4	5·5, 15·8	
Obesity grade 3	3	2·4	0·8, 6·7	3	2·4	0·8, 6·7	
DBM in households (*n* 109)							
DBM	9	10·8	4·8, 18·1	11	13·3	6·0, 20·5	0·3†

WHZ, weight-for-height-*Z*; HAZ, height-for-age-*Z*; UHNS-2019, Urban Health and Nutrition Study 2019; DBM, double burden of malnutrition.

* Paired *t* test.

† McNemar test.

‡ McNemar–Bowker test.

**Fig. 2:**
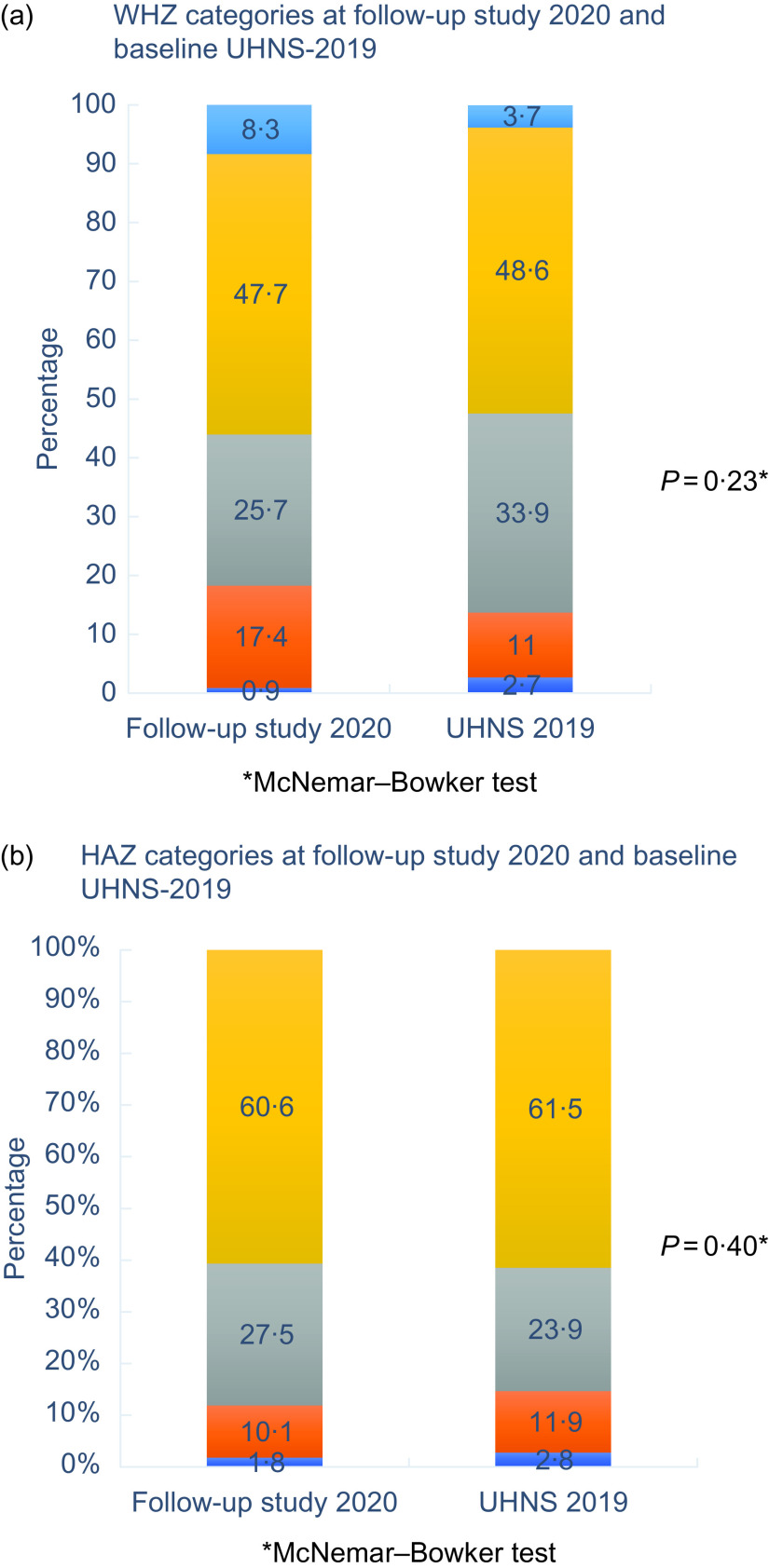
WHZ and HAZ categories at follow-up study in 2020 September and baseline UHNS-2019. WHZ, weight-for-height-*Z*; HAZ, height-for-age-*Z*; UHNS-2019, Urban Health and Nutrition Study 2019

When the WHZ and HAZ categories were compared between the follow-up and baseline studies (Fig. 2), there is an increase in the proportion of children with moderate wasting and overweight categories (Fig. 2(a)).

The proportion of children with adequate height has not changed and more children have moved from severe and moderate stunting to the mild stunting category (Fig. 2(b)).

None of the residents of selected households had COVID-19 infection and none were quarantined during the first 6-month period after the first COVID-19 case was reported (Table 3).

**Table 3:** Exposure to COVID-19 infection, food security status, changes in household income and expenditure during the COVID-19 pandemic (*n* 207)

Characteristic	Follow-up study, September 2020		UHNS-2019, September 2019		*P*
	*n*	%	*n*	%	
Exposure to COVID-19 infection					
Infected	0	0·0	–	–	
Quarantined	0	0·0	–	–	
Food security status					
Severe food insecurity	12	5·8	24	11·6	<0·001*
Moderate food insecurity	50	24·2	30	14·5	
Mild food insecurity	82	39·6	35	16·9	
Food secure	63	30·4	117	57·0	
Main coping mechanisms					
Sold household or productive assets	46	22·2	35	16·9	0·16†
Reduced non-food cost (education, health, etc)	55	26·6	24	11·6	<0·001†
Spent savings	100	48·3	44	21·3	<0·001†
Borrowed money from bank	42	20·3	29	14·0	0·1†
Borrowed food/money from friends/relatives	105	50·7	48	23·2	<0·001†
Looked for additional income sources	35	16·9	23	11·1	0·1†
Per capita monthly income (USD)					
< 27	50	24·2	23	11·1	<0·001*
27–41	53	25·6	43	20·8	
40·1, 59	53	26·1	67	32·9	
> 59·1	51	24·2	73	35·3	
Change in household income during last 6 months					
Increased	2	1·0	–	–	
Decreased	187	90·3	–	–	
No change	18	8·7	–	–	
Reasons to decrease household income during last 6 months (*n* 187)					
Loss of employment	121	64·7	–	–	
No extra income	75	40·1	–	–	
Deduct the salary by employee	92	49·2	–	–	
Change in household expenditure during last 6 months					
Increased	110	53·1	–	–	
Decreased	33	15·9	–	–	
No change	64	30·9	–	–	
Reasons to increase household expenditure during last 6 months (*n* 110)					
High food price	109	99·1	–	–	
Paid school fees	32	29·1	–	–	
Paid loans/vehicle lease	30	27·3	–	–	
Medicine cost	27	24·5	–	–	

UHNS-2019, Urban Health and Nutrition Study 2019

* McNemar–Bowker test.

† McNemar test.

Food security and coping strategies of selected households were compared between the two time periods; at baseline UHNS-2019 and the current follow-up study 1 year later (Table 3). Respondents were asked to focus on the preceding 6 months during which the COVID-19 pandemic occurred in Sri Lanka. Nearly half of the food-secure households in the UHNS-2019 were moved into the food insecurity category resulting in a total of two out of three households with food insecurity in the follow-up study. Nevertheless, the severe food-insecure population has reduced compared to last year (11·6 % *v*. 5·8%).

The most common coping strategies that were followed for food security needs were borrowing money from friends and use of savings, which was significantly increased in the follow-up study compared to baseline (50·7 %–23·2 %; *P* < 0·001 and 48·3 %–21·3 %; *P* < 0·001, respectively).

About 90 % of households reported a reduction in monthly household income during the preceding 6 months. Main contributory factors for reduced income were loss of employment (64·7 %), deduction of salary by the employer (49·2 %) and loss of extra income source (40·1 %).

Over 50 % of households reported increased household expenditure and almost all of these households (99·1 %) reported an increase in food prices as the main reason.

Highest prevalence of wasting, stunting and concurrent WaSt was observed in children between 24 and 35 months, <2500 *g* of birth weight and from severely food-insecure households (Table 4).

**Table 4:** Prevalence of wasting (moderate to severe), stunting (moderate to severe) and concurrent wasting and stunting (WaSt) by background characteristics in the follow-up study 2020

Variables	Wasting	Stunting	WaSt
	%			*n*
Age of the child in months				
12–23	13·6	4·5	4·5	22
24–35	23·3	23·3	13·3	30
36–47	20·8	4·2	4·2	24
48–59	12·5	12·5	0·0	16
≥ 60	18·8	12·5	5·9	17
Sex of the child				
Male	19·0	12·7	6·2	63
Female	17·4	10·9	6·5	46
Birth weight of the child (*g*)				
< 2500	35·3	17·6	11·1	17
2500–3000	14·3	11·9	4·8	41
> 3000	14·9	10·6	6·4	51
Current wasting of children				
Severe (<−3 sd)	–	**100·0	–	1
Moderate (−3 sd to −2 sd)	–	26·3	–	19
Mild (−2 sd to −1 sd)	–	3·6	–	28
Adequate (>−1 sd)	–	9·8	–	61
Wasting of children 1 year back				
Severe (<−3 sd)	***66·7	33·3	**33·3	3
Moderate (−3 sd to −2 sd)	75·0	25·0	25·0	12
Mild (−2 sd to −1 sd)	16·2	10·8	2·7	37
Adequate (>−1 sd)	5·3	8·8	3·4	58
Current stunting of children				
Severe (<−3 sd)	**0·0	–	–	2
Moderate (−3 sd to −2 sd)	54·5	–	–	11
Mild (−2 sd to −1 sd)	23·3	–	–	30
Adequate (≥−1 sd)	10·6	–	–	66
Stunting of children 1 year back				
Severe (<−3 sd)	**33·3	***100·0	***33·3	3
Moderate (−3 sd to −2 sd)	46·2	69·2	30·8	13
Mild (−2 sd to −1 sd)	26·9	3·8	3·8	26
Adequate (≥−1 sd)	9·0	0·0	1·5	68
Current food security status				
Food secure	21·2	11·1	8·8	63
Mild food insecurity	14·3	6·2	4·8	82
Moderate food insecurity	15·4	22·2	3·8	50
Severe food insecurity	37·5	8·3	12·5	12
Food security 1 year back				
Food secure	20·6	12·1	7·8	117
Mild food insecurity	6·2	9·5	0·0	35
Moderate food insecurity	16·7	11·5	5·6	30
Severe food insecurity	25·0	25·0	8·3	24
Current household per capita income (USD)				
< 27	22·6	16·1	9·7	50
27–41	13·8	6·9	0·0	53
40·1–59	22·6	12·9	6·5	53
> 59·1	11·1	11·1	10·5	51
Household per capita income 1 year back (USD)				
< 27	20·0	20·0	10·0	23
27–41	21·4	14·3	7·1	43
40·1–59	21·4	11·9	9·3	67
> 59·1	10·3	6·9	0·0	73
Pattern of household income during last 6 months				
Increased	0·0	0·0	0·0	2
Decreased	19·4	13·3	7·1	187
No change	10·0	0·0	0·0	18
Pattern of household expenditure during last 6 months				
Increased	20·3	15·6	9·2	110
Decreased	18·8	12·5	6·2	33
No change	13·8	3·4	0·0	64
Total	18·3	11·9	5·5	109

** *P* < 0·01.

*** *P* < 0·001.

The prevalence of wasting was significantly higher in children who are in the moderately stunted category in the present study, as well as in those who were in moderately wasted and moderately stunted categories 1 year ago. Significant differences were not seen in age, sex, food security, per capita income and wasting at present compared to 1 year ago.

The prevalence of stunting was significantly higher in severely wasted children at present compared to 1 year ago. Age, sex, per capita income, food security at present and 1 year ago were not significantly associated with the current stunting of children.

The prevalence of concurrent WaSt is 5·5 % and it is significantly higher in severely wasted and severely stunted children 1 year ago.

## Discussion

This study investigated the impact of COVID-19 pandemic on wasting, stunting, overweight/obesity and concurrent WaSt in children, obesity in women and food insecurity and double burden of malnutrition of households in underserved urban settlement areas in Colombo. Child wasting is an acute transient condition amenable to treatment with appropriate nutrition and medical interventions. Child stunting is chronic in nature and needs to be prevented^(17)^. The prevalence of concurrent WaSt among children under 5 years of age is an important indicator in child survival^(18)^. All three indicators are important in an emergency situation. Studies on this topic related to COVID-19 pandemic are limited, but a few available studies in other countries depict weight gain or no shift in weight^(19)^. Child wasting, stunting and concurrent WaSt provide wide range of information related to disruption of livelihood in this population due to COVID-19 pandemic. This study is a good example to show how marginalised populations will get affected further. As children are most vulnerable among low-income groups, focusing on child nutrition is needed in this type of population to prevent child malnutrition becoming a public health problem. We must consider these vulnerable groups while adopting universal COVID-19 prevention strategies overall.

In the current study, mean WHZ and HAZ of children have significantly improved over 1 year. However, the prevalence of wasting has increased from 13·8 % to 18·3 % (*P* = 0·26). Current wasting prevalence is above both the national average of 15·3 % and WHO critical level threshold of 15·0 %^(20)^. Although the risk of COVID-19 complications may be lower in children, the potential impact of reduced immunity associated with wasting should not be underestimated in this population^(21)^. Furthermore, it poses a grave concern due to the protracted course of the COVID-pandemic.

A positive change that was observed in this population was the reduction of child stunting, which indicates that shortage of food has not still affected the linear growth of children. However, monitoring of stunted children is important to prevent the long-term consequences during the COVID-19 pandemic due to its uncertain duration. Global experts predict an increased risk in all forms of malnutrition due to economic and food crises during periods of lockdown^(22)^.

It was alarming to observe the increasing number of overweight children in this population. Consuming cheaper products in greater quantities, increased consumption of unhealthy food due to limited choices and increased sedentary behaviour during lockdown periods due to limited space in their home settings are possible contributory factors. When the playgrounds in the neighbourhood were closed off and they were mandated to stay at home continuously for 52 d by the lockdown, children were more susceptible for weight gain. The closure of preschools which extended even beyond the lockdown period resulted in the absence of organised physical activity sessions for the young children. Furthermore, increased screen time that is also associated with increase in snacking, especially most available convenient food like sugary biscuits may be another contributory factor for the increase in overweight. Consistent findings have been reported in other countries^(23,24)^.

However, a change in obesity was not observed in women during the past year. This may become aggravated due to restrictions imposed on movement and changes in dietary habits due to economic hardships until solution is available for the COVID-19 pandemic. Considering the high level of obesity and 2·4 % morbid obesity in women, continuous monitoring is needed to prevent complications and increased susceptibility to this infection.

A worsening trend in food insecurity was observed in this population. The most probable reason could be frequent disruptions to their livelihoods due to repeated mandatory lockdowns to prevent disease transmission. These results further indicate that the pandemic has affected all income groups, which impacts the purchasing power of a household, worsening food insecurity. FIES may be a useful tool to measure trends and severity of food insecurity during COVID-19 pandemic, as it is based on participant responses to eight questions regarding constraints on their ability to obtain adequate food^(13)^.

To cope with a reduced income, participants have increased rate of borrowing, which was also observed in the UHNS-2019 study. Similar observations were made in other populations whose earning opportunities were abandoned^(25–28)^. Other coping strategies that were observed in the current study include taking loans and selling assets, which will exacerbate poverty and inadvertently affect child stunting in the future. All these indicate a weakened resilience in USS in Colombo due to the COVID-19 pandemic.

Our study further shows that stunted children are more likely to become severely wasted and severely wasted children are more likely to develop concurrent WaSt. Early detection and appropriate treatment of concurrent WaSt should be prioritised and steps should be taken via the health care system to minimise its impact on child survival.

Similar to other studies, our study suggests that severe wasting in children can lead to stunting^(18)^. Furthermore, two-thirds of severely wasted children in the past year remain wasted indicating the importance of revisiting existing intervention programmes. However, it should be interpreted with caution due to small sample size and studies with larger sample sizes are needed to confirm it. Concurrent WaSt deserves more attention during the COVID-19 pandemic because of its impact on child survival^(18)^. This study indicates that presence of severe stunting and severe wasting in the past are good indicators to identify high-risk children for concurrent WaSt if these children have not attended routine growth monitoring clinics due to lockdown. The lack of a significant association between household food insecurity and child malnutrition in our study implies that merely improving household food security may not be sufficient to improve the nutritional status of children in this population. Therefore prevention, early detection and appropriate treatments are needed for children with wasting, stunting and concurrent WaSt to minimise their susceptibility to the COVID-19 infection.

This is the first follow-up study in Sri Lanka to assess the impact of COVID-19 on nutrition status and food security in USS. The main strength of this study revolves around repeated measurements of same households and individuals by the same research team. However, the small sample size is a limitation as we had to minimal interactions with households due to social restrictions imposed during the pandemic. Access and coverage of nutrition services were not assessed in this study. Since this was a rapid assessment with short contact time with the study participants, we did not collect information on any food assistance received and access and coverage of nutrition services which could have better explained the findings.

## Conclusions

In underserved urban settlements of Sri Lanka, there is an increase in the prevalence of child wasting and overweight after the first wave of COVID-19 pandemic. Overall child wasting is alarmingly high and we recommend providing supplementary food for children less than 5 years of age while paying attention to overweight children. There is an urgent need to focus on morbidly obese women to minimise their susceptibility to COVID-19 and to control unhealthy weight gain of women to prevent further increase in obesity. This population needs to be monitored for food security on a long-term basis as repeated lockdowns may worsen the outcome.
